# Detecting muscle fatigue during lower limb isometric contractions tasks: a machine learning approach

**DOI:** 10.3389/fphys.2025.1547257

**Published:** 2025-03-13

**Authors:** Jiaqi Sun, Cheng Zhang, Guangda Liu, Wenjie Cui, Yubing Sun, Chunyan Zhang

**Affiliations:** ^1^ College of Instrumentation and Electrical Engineering, Jilin University, Changchun, China; ^2^ Department of Rehabilitation Medicine, First Hospital of Jilin University, Changchun, China

**Keywords:** machine learning, isometric contractions, ICEEMDAN, t-SNE, muscle fatigue

## Abstract

**Background:**

Muscle fatigue represents a primary manifestation of exercise-induced fatigue. Electromyography (EMG) serves as an effective tool for monitoring muscle activity, with EMG signal analysis playing a crucial role in assessing muscle fatigue. This paper introduces a machine learning approach to classify EMG signals for the automatic detection of muscle fatigue.

**Methods:**

Ten adult participants performed isometric contractions of lower limb muscles. The EMG signals were decomposed into multiple intrinsic mode functions (IMFs) using improved complementary ensemble empirical mode decomposition adaptive noise (ICEEMDAN). Time-domain, frequency-domain, time-frequency domain, and nonlinear features associated with muscle fatigue during isometric contraction were analyzed through EMG signals. Dimensionality reduction was achieved using t-distributed stochastic neighbor embedding (t-SNE), followed by machine learning-based classification of fatigue levels.

**Results:**

The findings indicated that EMG signal characteristics changed significantly with increasing fatigue. The combination of support vector machines (SVM) and ICEEMDAN achieved an impressive accuracy of 99.8%.

**Conclusion:**

The classification performance of this study surpasses that of existing state-of-the-art methods for detecting exercise-induced fatigue. Therefore, the proposed strategy is both valid and effective for supporting the detection of muscle fatigue in training, rehabilitation, and occupational settings.

## 1 Introduction

Muscle fatigue is a critical component of exercise-induced fatigue and serves as a primary indicator of muscle performance and endurance during physical activities (Behm et al., 2021). Muscle fatigue results in diminished exercise capacity, characterized by reduced muscle strength and decreased power output (Supruniuk et al., 2023). Lower limbs play a key role in the exercise process (Finn et al., 2020; Kulmala et al., 2020). Numerous researchers have employed various techniques to analyze lower limb movement (Fidalgo-Herrera et al., 2021; Mehmood et al., 2021; Zhang W. B. et al., 2024). In muscle submaximal tasks, fatigue manifests as increased neural drive to offset peripheral declines in muscle force capability, as evident by concomitant measurements of EMG and motor pathway excitability (Ruggiero and McNeil, 2019). Effective monitoring and assessment of muscle fatigue are essential for optimizing training protocols, preventing injuries, and enhancing performance in sports and rehabilitation settings.

Electromyography (EMG) has emerged as a valuable tool for real-time monitoring and analysis of muscle activity (Rampichini et al., 2020; Hou et al., 2021; Sun et al., 2022). By recording the electrical signals generated by muscle fibers during contraction, EMG enables researchers and practitioners to evaluate muscle function and detect early signs of fatigue.

In recent years, there has been a growing interest in leveraging machine learning techniques to automate the detection and classification of muscle fatigue based on EMG signals (Makaram et al., 2021; Liu et al., 2023). By extracting relevant features from EMG data and training classification models, these approaches provide a data-driven framework for objectively assessing fatigue levels. Studies on lower limb rehabilitation training using EMG primarily focus on time-frequency domain analysis (Hatamzadeh et al., 2023; Daniel et al., 2024). Given the non-stationary and nonlinear characteristics of EMG signals (Wang S. R. et al., 2021). Nonlinear dynamic analysis can better decode the variation trends during muscle fatigue (García-Aguilar et al., 2022).

This study aims to propose a novel machine learning strategy based on the classification of electromyography (EMG) signals for the automatic detection of muscle fatigue. Specifically, we focus on isometric contractions of lower limb muscles, a common scenario in both athletic training and rehabilitation programs. The proposed methodology incorporates advanced signal processing techniques, including improved complementary ensemble empirical mode decomposition adaptive noise (ICEEMDAN) for decomposing EMG signals into intrinsic mode functions (IMFs) (Tian et al., 2024).

We conduct a comprehensive analysis of the time-domain, frequency-domain, time-frequency domain, and nonlinear features extracted from EMG signals to characterize muscle fatigue during isometric contractions. Additionally, we employ t-distributed stochastic neighbor embedding (t-SNE) for feature dimensionality reduction, facilitating a more concise representation of the data (Olobatuyi et al., 2024).

Machine learning algorithms, particularly Support Vector Machines (SVM), are employed to classify fatigue levels based on the extracted features (Chauhan et al., 2019). Our results demonstrate significant changes in EMG features as fatigue levels increase, underscoring the potential of EMG-based classification for fatigue assessment.

Notably, the combination of SVM and ICEEMDAN achieves an impressive classification accuracy of 99.8%, surpassing existing methods for exercise fatigue classification (Wang J. H. et al., 2021). This study significantly enhanced the accuracy and efficiency of muscle fatigue detection by introducing advanced signal processing and machine learning techniques, providing new ideas and methods for research and application in related fields, including training, rehabilitation, and occupational health.

## 2 Materials and methods

**FIGURE 1:**
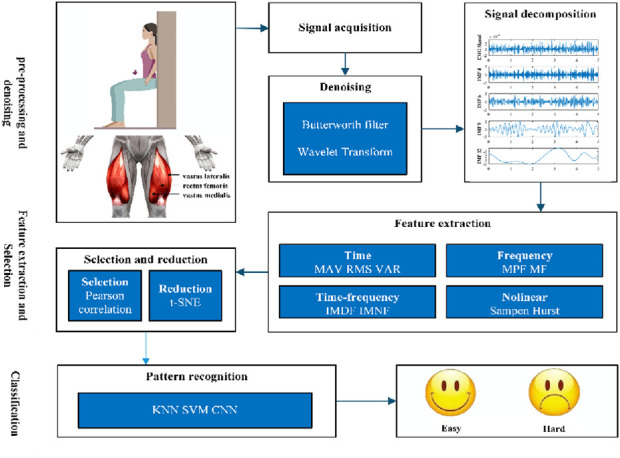
Methodology proposed for the implementation of the exercise fatigue classifier.

The proposed methodology aims to develop the classification system for assessing exercise fatigue of three general phases: 1) the preprocessing, filtering, and decomposition of the signals; 2) the extraction and reduction of features; and 3) the classification of the data (Figure 1).

The subsequent sections detail each phase and outline the metrics essential for developing and evaluating the classifier.

### 2.1 Data acquisition

Lower extremity muscle isometric contraction fatigue in the subjects was induced by performing wall squats. The American Delsys Trigno electromyography system collected signals from the quadriceps muscles, including the vastus medialis, rectus femoris, and vastus lateralis. The data collection frequency was 1000 Hz. The area was first cleaned with mild soap and water to remove surface contaminants. Excessive hair was trimmed or shaved to ensure adequate electrode contact. An abrasive gel (e.g., NuPrep) was applied and gently rubbed in circular motions for approximately 15–20 s to remove dead skin cells and reduce impedance. The area was then wiped with an alcohol swab to remove residual gel and dried thoroughly. Surface electrodes were strategically positioned over the muscle bellies of interest, with specific locations determined based on standardized anatomical landmarks. During the test, subjects were asked to subjectively rate their perceived level of fatigue as either easy or difficult. This study was approved by the Ethics Committee of Jilin University and conforms to the Helsinki Declaration.

Participants were healthy individuals with no known diseases and had not consumed alcohol or drugs prior to the experiment. They read and signed an informed consent form before participating. The experiment was conducted under the supervision of a tester, with subjects instructed to warm up before the test and stop immediately if they felt any discomfort during the procedure. During the test, subjects performed wall squats with their backs against the wall, legs naturally spaced shoulder-width apart, and arms relaxed at their sides. The torso was positioned at a 90-degree angle to the thighs, and the thighs were at a 90-degree angle to the calves. Subjects continued the task until exhaustion. A total of ten participants completed the experiment, each performing ten trials, resulting in a total of 300 complete test datasets. The basic information for ten male participants in the study is shown in Table 1.

**TABLE 1:** Descriptive data of participants.

Variable	Mean (SD)
Age (years)	24.21 ± 3.65
Height (cm)	169.58 ± 4.53
Weight (kg)	62.88 ± 9.22
Body Mass Index (kg/m^2^)	21.86 ± 3.62

### 2.2 Pre-processing

EMG signals are inherently susceptible to noise and artifacts, which can arise from various sources such as electrical interference, motion artifacts, and physiological factors (Ashraf et al., 2023; Boyer et al., 2023). These extraneous components can obscure the underlying muscle activity patterns, thereby impeding accurate interpretation of the EMG signals. Consequently, the implementation of effective preprocessing techniques is essential for enhancing the quality and reliability of EMG signal analysis. Bandpass filtering selectively attenuates noise outside the frequency range of interest, while high-pass filtering removes low-frequency drift and motion artifacts. A notch filter specifically targets and eliminates 50 Hz power line interference. Additionally, baseline correction is imperative to remove baseline drift and establish a consistent reference level for EMG signals. Baseline drift elimination is accomplished through mean subtraction, which involves subtracting the mean value of the signal from each data point to center the signal around zero.

### 2.3 Signal decomposition

The recorded EMG signals often contain redundant information, making direct classification from the raw data suboptimal. To extract meaningful features for signal classification, it is more effective to decompose the signal into multi-resolution components that capture relevant information about the muscle state. ICEEMDAN represents an advanced signal processing technique that builds upon the traditional Empirical Mode Decomposition (EMD) method. EMD, initially developed by Huang et al. in the late 1990s, was designed to decompose non-stationary and nonlinear signals into a finite number of oscillatory components known as IMFs (Colominas et al., 2014). However, EMD is subject to certain limitations, including mode mixing and sensitivity to noise, which can significantly impede its effectiveness, particularly in practical applications. To mitigate these challenges, ICEEMDAN incorporates several enhancements. It addresses the inherent limitations of EMD by introducing adaptive noise and ensemble strategies, thereby improving its performance in handling non-stationary and noisy signals. At its core, ICEEMDAN decomposes a given signal into a finite number of IMFs and a residual component. What distinguishes ICEEMDAN is its adaptive noise mechanism, which dynamically adjusts to the characteristics of the signal during the decomposition process. This adaptability enables ICEEMDAN to effectively extract meaningful components from signals corrupted by varying degrees of noise, a common issue in real-world signal processing applications. Additionally, ICEEMDAN employs an ensemble strategy, where multiple decompositions are performed with randomized initialization conditions. By aggregating the results from these ensemble decompositions, ICEEMDAN enhances the robustness of the decomposition process and minimizes the risk of obtaining biased results.

ICEEMDAN algorithm is described as follows (Bommidi et al., 2023).

Step 1: Noise addition by Formula 1:

$$x\left(i\right)=x+\beta_{0}E\left[w^{\left(\mathrm{i}\right)}\right]$$ (1)

where x is the original signal, β^0^ is the noise standard deviation, $E\left(w^{\left(\mathrm{i}\right)}\right)$ is the special noise, $w^{\left(\mathrm{i}\right)}$ is the added *i*th Gaussian white noise.

Step 2: EMD decomposition by Formulas 2, 3:

$$r_{1}=\frac{1}{N}\sum_{i=1}^{N}M\left[x\left(i\right)\right]$$ (2)

$${\tilde{c}}_{1}=x-r_{1}$$ (3)

where $r_{1}$ is the residual of the first decomposition, $M\left[\cdot \right]$ is the operator to compute the local mean and ${\tilde{c}}_{1}$ is the value of the first IMF.

Step 3: Iterative decomposition by Formulas 4, 5:

$$r_{k}=\frac{1}{N}\sum_{i=1}^{N}M\left\{r_{k-1}+\beta_{k-1}E_{k}\left[w^{\left(i\right)}\right]\right\},\;k=2,3,\ldots N$$ (4)

$${\tilde{c}}_{k}=r_{k-1}-r_{k}$$ (5)

where $r_{k}$ is the residual of the *k*th decomposition and ${\tilde{c}}_{k}$ is the value of the *k*th IMF.

The versatility of ICEEMDAN makes it a valuable tool across various domains, including biomedical signal processing, financial forecasting, environmental monitoring, and structural health monitoring. Its ability to effectively handle noisy and non-stationary signals through adaptive noise modulation and ensemble decomposition makes it particularly useful in applications where precise signal analysis is crucial for decision-making and inference.

### 2.4 Feature extraction

Effective feature extraction is crucial for deriving meaningful information from EMG signals and elucidating muscle activation patterns. Feature extraction serves a pivotal role in quantifying various aspects of muscle activity, including intensity, timing, frequency content, and temporal dynamics. This section provides an overview of commonly employed EMG feature extraction methods and their applications in muscle activity analysis.

#### 2.4.1 Time domain features

Time domain features capture the amplitude and temporal characteristics of EMG signals. Commonly extracted features include Mean Absolute Value (MAV), Variance (VAR), and Root Mean Square (RMS) (Chen et al., 2020; Wang H. B. et al., 2021) according to Formulas 6–8. MAV provides information on the overall magnitude of the signal. VAR indicates the variability or fluctuations within the signal. RMS reflects the overall energy or power of the signal.

$$\text{MAV}=\frac{1}{N}\sum_{i=1}^{N}\;\left|x_{i}\right|$$ (6)

$$\text{VAR}=\frac{1}{N}\sum_{i=1}^{N}\;{\left(x_{i}-\bar{x}\right)}^{2}$$ (7)

$$\text{RMS}=\sqrt{\frac{1}{N}\sum_{i=1}^{N}\;x_{i}^{2}}$$ (8)

where $x_{i}$ represents the EMG signal samples and *N* is the total number of samples in the window. $\bar{x}$ is the mean of the EMG signal.

#### 2.4.2 Frequency domain features

Frequency domain analysis of EMG signals enables the characterization of spectral content and frequency distribution. Mean Frequency (MF) and Median Frequency (MPF) are commonly used features to describe the central tendency of frequency components in the signal (Zhang X. D. et al., 2024) according to Formulas 9, 10. MF provides information on the central tendency of the frequency distribution, while MPF serves as a measure of the signal’s spectral center of mass.

$$\text{MF}=\frac{\sum_{i=1}^{N}\;f_{i}P_{i}}{\sum_{i=1}^{N}\;P_{i}}$$ (9)

$$\text{MPF}==\frac{1}{2}\sum_{i=1}^{N}\;P_{i}$$ (10)

where $f_{i}$ is the frequency and $P_{i}$ is the power at that frequency.

#### 2.4.3 Time-frequency domain features

Time-frequency domain analysis captures both temporal and spectral characteristics of EMG signals. Instantaneous Mean Frequency (IMDF) and Instantaneous Median Frequency (IMNF) provide insights into the dynamic changes in frequency content over time (Ghosh and Swaminathan, 2020). IMDF represents the mean frequency content of the signal at various time points, capturing how the frequency content evolves over time. IMNF, similar to IMDF, represents the median frequency content at different time points, offering insight into the temporal variations of the signal’s spectral characteristics.

#### 2.4.4 Non-linear feature

EMG signals exhibit complex dynamics that cannot be fully captured by linear methods alone. Nonlinear feature extraction aims to capture the intricate patterns, irregularities, and nonlinear interactions within the signal. Sample Entropy quantifies the regularity or predictability of the EMG signal (Chatain et al., 2020). It measures the likelihood that similar patterns of data points will repeat over a specified length within the signal. The Hurst exponent is a measure of long-range dependence or self-similarity in the EMG signal (Irfan et al., 2023). It characterizes the persistence or anti-persistence of trends in the signal across different time scales.

### 2.5 Feature reduction

The features of EMG signals during exercise were extracted, and a dimensionality reduction was performed to map the selected feature vectors into a lower-dimensional space. Linear methods assume that the underlying relationships between variables are linear, which may not hold true for all datasets. Consequently, linear techniques may fail to capture complex, non-linear relationships present in the data. Although linear methods preserve most of the variance, they may not retain all the information inherent in the original high-dimensional space. Non-linear techniques, on the other hand, can capture complex relationships and manifold structures in the data that linear methods may overlook. Methods such as t-SNE excel at preserving local structures, making them particularly suitable for tasks like visualization. Non-linear techniques effectively handle high-dimensional data by capturing its intrinsic structure.

t-SNE (t-Distributed Stochastic Neighbor Embedding) is a dimensionality reduction technique primarily used for visualizing high-dimensional data in a lower-dimensional space, typically 2D or 3D. The principle behind t-SNE involves mapping multi-dimensional data points to a lower-dimensional space while preserving their pairwise similarities as accurately as possible. The process begins by computing pairwise similarities between data points in the high-dimensional space. These similarities are typically measured using Gaussian kernels based on the Euclidean distance between points, which quantifies how close or similar each pair of points is in the original space. Next, t-SNE constructs a similar map in the lower-dimensional space (e.g., 2D or 3D) by placing points such that similar points in the original space remain close together in the new space. The positions of points in the lower-dimensional space are optimized iteratively to minimize the discrepancy between the original pairwise similarities and the similarities in the lower-dimensional space. The optimization process in t-SNE minimizes the Kullback-Leibler (KL) divergence between the distribution of pairwise similarities in the original space and the distribution of pairwise similarities in the lower-dimensional space. This divergence measurement ensures that similar points are represented by nearby points in the lower-dimensional embedding. t-SNE introduces a hyperparameter called perplexity, which balances the attention given to local *versus* global aspects of the data. It influences the number of nearest neighbors considered when computing similarities. A higher perplexity value results in a more global view of the data, while a lower value focuses more on local relationships. The optimization of t-SNE is performed using gradient descent methods to minimize the KL divergence. It adjusts the positions of points in the lower-dimensional space iteratively until convergence criteria are met. Unlike linear methods like PCA (Principal Component Analysis), t-SNE is inherently non-linear and is particularly effective at capturing complex structures and relationships in high-dimensional data, such as clusters, nonlinear manifolds, and local neighborhoods.

The t-SNE algorithm is as follows.

Step 1 Similarity in High-Dimensional Space by Formula 11:

In the high-dimensional space, the similarity between data points $x_{i}$ and $x_{j}$ is measured using a Gaussian distribution:

$$p_{j|i}=\frac{\exp\left(-\frac{{\left\|x_{i}-x_{j}\right\|}^{2}}{2\sigma_{i}^{2}}\right)}{\sum_{k\neq i}\;\exp\left(-\frac{{\left\|x_{i}-x_{k}\right\|}^{2}}{2\sigma_{i}^{2}}\right)}$$ (11)

Here, $\sigma_{i}$ is the standard deviation of the Gaussian distribution for point $x_{i}$ , which controls the perplexity (a measure of the effective number of neighbors). $p_{j|i}$ represents the probability that $x_{j}$ is a neighbor of $x_{i}$ in the high-dimensional space.

To make the similarity matrix symmetric, a joint probability distribution $p_{ij}$ is defined by Formula 12:

$$p_{ij}=\frac{p_{j|i}+p_{i|j}}{2N}$$ (12)

where *N* is the total number of data points.

Step 2 Similarity in Low-Dimensional Space by Formula 13:

In the low-dimensional space, the similarity between mapped points yi and yj is measured using a Student’s t-distribution with one degree of freedom (which is equivalent to a Cauchy distribution):

$$q_{ij}=\frac{{\left(\left.1+\|\right\|yi-yj{\|}^{2}\right)}^{-1}}{\sum_{k\neq l}\;{\left(\left.1+\|\right\|yk-yl{\|}^{2}\right)}^{-1}}$$ (13)

The t-distribution has a heavier tail compared to the Gaussian distribution, which helps to alleviate the “crowding problem” in low-dimensional space.

Step 3 Optimization Objective by Formula 14:

The goal of t-SNE is to minimize the Kullback-Leibler (KL) divergence between the joint probability distributions *P* and *Q* in the high-dimensional and low-dimensional spaces, respectively:

$$C=\overset{\;}{\underset{i}{\sum }}KL\left(P_{i}\|Q_{i}\right)=\overset{\;}{\underset{i}{\sum }}\overset{\;}{\underset{j}{\sum }}p_{ij}\log\frac{p_{ij}}{q_{ij}}$$ (14)

$P_{i}$ and $Q_{i}$ are the conditional probability distributions for point i in the high-dimensional and low-dimensional spaces, respectively. The KL divergence measures the difference between the two probability distributions, and the optimization aims to make *P* and *Q* as close as possible.

Step 4 Gradient Descent Update by Formula 15:

To minimize the KL divergence, the low-dimensional embeddings $y_{i}$ are updated using gradient descent:

$$\frac{\partial C}{\partial y_{i}}=4\overset{\;}{\underset{j}{\sum }}\;\left(p_{ij}-q_{ij}\right)\left(y_{i}-y_{j}\right)$$ (15)

In summary, t-SNE transforms high-dimensional data into a lower-dimensional representation while preserving local and some global structures by minimizing the Kullback-Leibler (KL) divergence between the pairwise similarities of the original and lower-dimensional spaces. It is widely employed for exploratory data analysis, visualization, and understanding complex data patterns in fields such as machine learning, data mining, and natural language processing.

### 2.6 Classification

Machine learning classifiers learn patterns from labeled data to predict the labels of new, unseen data points. Machine learning classification methods aim to construct models that can automatically categorize data into predefined classes or categories. These methods can be broadly categorized into two main types: generative and discriminative. Generative classifiers model the joint probability distribution of the features and class labels. They learn the probability distribution of each class and use Bayes' theorem to calculate the posterior probability of each class given the input features. Examples of generative classifiers include Naive Bayes, Gaussian Mixture Models (GMM), and Hidden Markov Models (HMM). Discriminative classifiers directly model the decision boundary that separates different classes in the feature space. They aim to find a function that maps input features to class labels without explicitly modeling the underlying probability distribution. Examples of discriminative classifiers include Logistic Regression, Decision Trees, Random Forests, SVM, and Neural Networks.

SVM is a powerful discriminative classifier designed to identify the optimal hyperplane that maximally separates different classes in the feature space while maximizing the margin between the hyperplane and the nearest data points from each class. SVMs excel in handling both linearly separable and non-linearly separable data by employing various kernel functions to map the data into higher-dimensional spaces, enabling linear separation. SVMs are particularly effective in high-dimensional spaces and are less prone to overfitting; however, they can be computationally intensive, especially for large datasets.

## 3 Results

**FIGURE 2:**
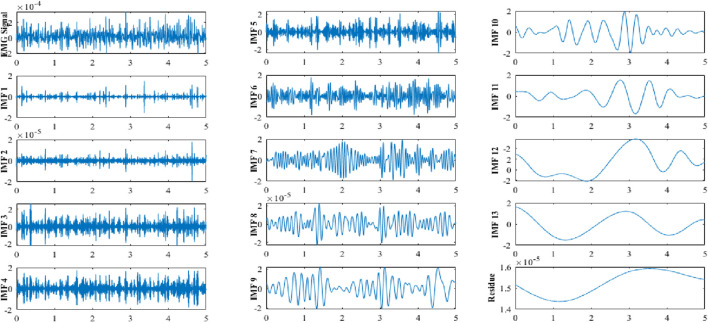
ICEEMDAN decomposition of an EMG signal in its 13 IMF_i_, i = (1,2, … ,13).

The results of our study demonstrate a comprehensive approach to fatigue classification utilizing electromyographic (EMG) signals. We began the process with preprocessing the EMG signals, followed by decomposition using ICEEMDAN. This decomposition facilitated the extraction of components in both the time and frequency domains, enabling a detailed analysis of the underlying physiological phenomena associated with fatigue. Figure 2 illustrates the decomposition of a preprocessed EMG signal into 13 IMFs and a residual component using the ICEEMDAN method. This figure demonstrates the effectiveness of ICEEMDAN as a standardized methodology for accurately decomposing signals.

**FIGURE 3:**
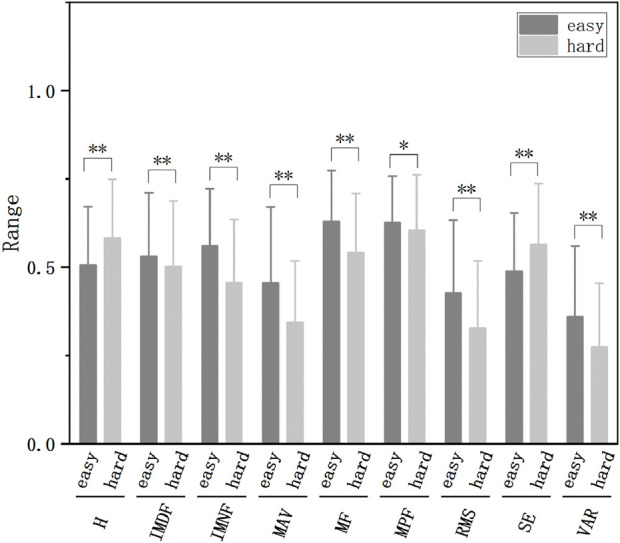
The normalized features of different fatigue levels extracted from raw data (*p < 0.05, **p < 0.01).

**FIGURE 4:**
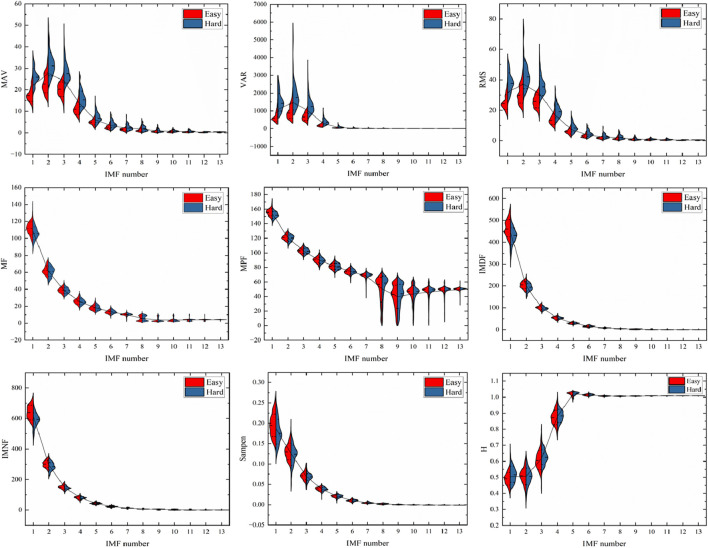
Changes in the features of the IMF of EMG during exercise.

Subsequently, we conducted feature extraction on the raw data and decomposed components, encompassing time-domain, frequency-domain, time-frequency domain, and nonlinear features. This multi-dimensional feature set captured diverse aspects of the signal characteristics associated with fatigue, thereby enhancing the discriminative power of our classification model. The trend changes of these features are illustrated in Figures 3, 4.

**FIGURE 5:**
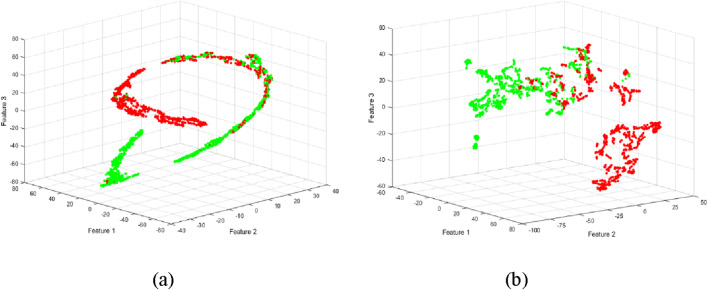
Scattergrams using t-SNE method for reducing the high dimensionality of the feature set. **(a)** Dimensionality reduction of features extracted from raw data. **(b)** Dimensionality reduction of features extracted from decomposed EMG data.

To visualize and further refine the feature space, we employed t-SNE to nonlinearly reduce the dimensionality of the extracted features to three dimensions. This transformation facilitated a more intuitive representation of the data, potentially aiding in the identification of distinct clusters corresponding to different fatigue levels. t-SNE dimensionality reduction was applied to both the original and decomposed EMG features, as illustrated in Figure 5.

**FIGURE 6:**
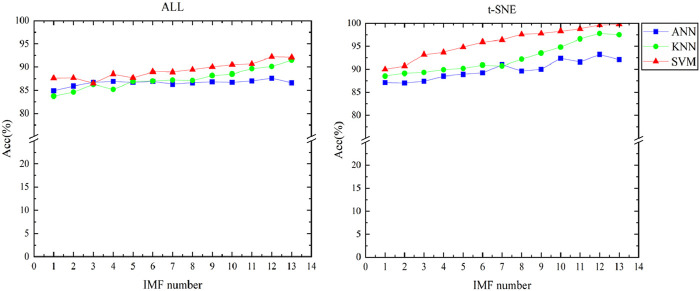
Classification accuracy of original feature and feature dimensionality reduction.

Finally, we employed three machine learning methods (ANN, KNN, SVM) for classification, leveraging the enriched feature set and the reduced feature space obtained through t-SNE. SVM exhibited the best performance. As illustrated in Figure 6, the classification accuracy increases with the number of IMFs, and the classification accuracy using dimensionality-reduced features is higher than that of the original features. Our findings demonstrate that, compared to the original features, the dimensionality-reduced features have markedly enhanced the classification performance. The method for calculating accuracy is as follows by Formula 16:

$$Accurary=\frac{TP+TN}{TP+TN+FP+FN}$$ (16)

where *TP* (True Positive) represents the number of true positive results, *TN* (True Negative) represents the number of true negative results, *FP* (False Positive) represents the number of false positive results, and *FN* (False Negative) represents the number of false negative results.

**FIGURE 7:**
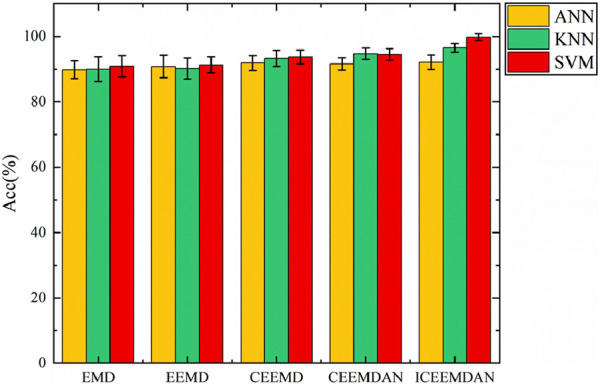
Comparison of the achieved classification accuracy obtained from five different signal decomposition methods.

The classification accuracies were achieved by combining three classifiers with EMD, Ensemble Empirical Mode Decomposition (EEMD), Complementary Ensemble Empirical Mode Decomposition (CEEMD), Complementary Ensemble Empirical Mode Decomposition with Adaptive Noise (CEEMDAN), and ICEEMDAN for predicting exercise-induced fatigue are noteworthy. The accuracy of classification results for various decomposition methods is shown in Figure 7. In comparing the decomposition methods, the three classifiers exhibit similar performance overall, with ICEEMDAN and SVM demonstrating the best combination. Specifically, SVM exhibited higher accuracy. EMD-based methods demonstrate promising results, with EMD alone achieving an accuracy of 90.9%, followed by EEMD at 91.3%. These methods leverage empirical mode decomposition to extract IMFs, which capture the underlying oscillatory modes present in the fatigue data. Moving towards enhanced performance, CEEMD and CEEMDAN further refine the decomposition process, leading to accuracies of 93.7% and 94.5%, respectively. These improvements can be attributed to the complementary advantages of ensemble empirical mode decomposition and complementary ensemble empirical mode decomposition with adaptive noise, which effectively handle nonlinearity and non-stationarity in the fatigue signals. Notably, ICEEMDAN achieves the highest accuracy of 99.8%, indicating its superior capability in fatigue classification when combined with SVM. ICEEMDAN integrates ensemble empirical mode decomposition with an adaptive noise algorithm, offering enhanced adaptability to complex signal variations and noise. ICEEMDAN effectively captures subtle variations in fatigue-related EMG signals, leading to superior classification performance.

## 4 Discussion

Muscle fatigue is a critical factor influencing physical performance, rehabilitation outcomes, and occupational safety (Sun et al., 2022). The ability to accurately detect muscle fatigue is essential for optimizing training regimens, preventing injuries, and enhancing the efficacy of rehabilitation programs. Traditional methods for assessing muscle fatigue often rely on subjective measures (Zhou et al., 2011) or invasive procedures, which may not provide real-time or precise feedback. This paper highlights the efficacy of integrating advanced signal processing techniques with machine learning algorithms for accurate and reliable fatigue classification, aiming to improve the accuracy and effectiveness of muscle activity analysis in various research and clinical settings.

The results of this study highlight the effectiveness of combining improved complementary ensemble empirical mode decomposition adaptive noise (ICEEMDAN) (Liang et al., 2022) with support vector machines (SVM) for classifying EMG signals. The decomposition of EMG signals into intrinsic mode functions (IMFs) using ICEEMDAN allows for a detailed analysis of muscle activity across multiple domains (time, frequency, time-frequency, and nonlinear). This comprehensive approach captures the nuances of muscle fatigue progression, which is reflected in the significant changes observed in EMG signal characteristics as fatigue increases. The use of t-distributed stochastic neighbor embedding (t-SNE) for dimensionality reduction further enhances the classification performance by preserving the local structure of the data while reducing its complexity. This superior performance can be attributed to the robustness of ICEEMDAN in handling non-linear and non-stationary EMG signals, combined with the powerful classification capabilities of SVM (Chauhan et al., 2019). The outstanding accuracy achieved by ICEEMDAN emphasizes its potential for real-world applications requiring highly accurate fatigue detection, such as sports performance monitoring and healthcare diagnostics. The superior performance of ICEEMDAN can be attributed to its ability to effectively capture subtle variations in fatigue-related EMG signals, which is crucial for distinguishing between different levels of fatigue. This capability is particularly valuable in dynamic environments where signal characteristics can vary significantly.

Our study demonstrates that the combination of ICEEMDAN and SVM provides a powerful tool for fatigue classification using EMG signals. Compared with the results of fatigue classification based on simple EMG characteristics, this study has a higher accuracy (Jamaluddin et al., 2017). Further investigations could focus on exploring the robustness of these methods across diverse fatigue datasets, including different types of physical activities and patient populations, and evaluating their generalization capabilities in real-world scenarios. Additionally, future work could involve exploring other advanced signal processing techniques and machine learning algorithms to further enhance the accuracy and reliability of fatigue classification. The integration of real-time data processing and adaptive learning mechanisms could also be explored to develop more responsive and personalized fatigue monitoring systems. In sports training, the ability to accurately detect muscle fatigue in real-time can help coaches and athletes optimize training intensity and duration, thereby reducing the risk of overtraining and injury. In rehabilitation settings, this method can provide objective feedback to clinicians, enabling them to tailor exercise protocols to individual patient needs and monitor progress more effectively. In occupational settings, particularly those involving repetitive or physically demanding tasks, the detection of muscle fatigue can help prevent work-related musculoskeletal disorders and improve worker safety.

## 5 Conclusion

In conclusion, our study demonstrates the efficacy of a multi-stage approach for identifying exercise-induced fatigue using electromyographic (EMG) signals. By preprocessing the EMG signals and decomposing them using ICEEMDAN, we were able to extract a comprehensive set of features encompassing time-domain, frequency-domain, time-frequency domain, and nonlinear characteristics from the resulting components. Furthermore, employing t-SNE enabled us to reduce the dimensionality of the extracted features to three dimensions, facilitating a more intuitive representation of the data. This nonlinear dimensionality reduction technique aided in capturing the intrinsic structure of the feature space, potentially improving the discriminative power of subsequent classification models. Finally, leveraging SVM for classification, we observed significantly enhanced classification performance compared to conventional methods. The integration of advanced signal processing techniques with machine learning algorithms, as demonstrated in our approach, holds promise for robust and accurate fatigue classification in various domains, including sports science, healthcare, and human-computer interaction.

Overall, our findings underscore the importance of adopting a holistic approach that integrates signal processing, feature extraction, dimensionality reduction, and classification techniques for effective fatigue recognition. Future research endeavors could investigate the generalizability and scalability of our methodology across diverse populations, activities, and real-world applications.

## Data Availability

The raw data supporting the conclusions of this article will be made available by the authors, without undue reservation.
